# Experiences and management of urinary incontinence following treatment for prostate cancer: Disrupted embodied practices and adapting to maintain masculinity

**DOI:** 10.1177/13634593231185266

**Published:** 2023-06-30

**Authors:** Richard Green

**Affiliations:** University of Surrey, UK

**Keywords:** embodiment, incontinence, masculinities, prostate cancer, stigma

## Abstract

This article explores men’s experiences of and management strategies for urinary incontinence (UI) following treatment for prostate cancer. Qualitative interviews with 29 men, recruited from two prostate cancer support groups, explored their post-treatment experiences. Drawing on a conceptual toolkit connecting theories of masculinities, embodiment, and chronic illness, this paper identifies older men’s experiences and strategies for managing UI and explores how these are shaped by their masculinities. This article identifies interdependence between managing stigma for UI and maintaining masculinity. Men’s embodied practices for engaging in activities in public, crucial to masculine identity, were disrupted. In response, they adopted new reflexive body techniques to manage and resolve their UI, and thereby address the threat to their masculine identities, characterised in three strategies: *monitoring, planning*, and *disciplining*. The new embodied practices men described suggest three factors as important components for adopting new reflexive body techniques: *routine, desire*, and *unruliness*.

## Stigma, urinary incontinence, and prostate cancer

A stigma is an aspect of a person that is socially ascribed as being discrediting (Goffman, 1963). Enacted stigma is the overt discrimination resulting from stigma because of its ‘social unacceptability’, whereas felt stigma is the feeling of shame or fear of potentially experiencing enacted stigma (Scambler, 2009). Goffman (1963) describes how being in a ‘discreditable’ state leaves people with the ongoing concern of how much information they want to give to others about their stigma. He suggests there are three main strategies for managing this: ‘passing, covering, and withdrawal’. Passing involves managing ‘undisclosed discrediting information about self’ (Goffman, 1963: 42). Covering involves managing being discredited when stigma is evident so that the stigma does not disrupt social encounters (Goffman, 1963: 102). Lastly, withdrawal is the limitation and sometimes cessation of social activities with others. Such strategies help stigmatised people to adjust others’ perceptions towards themselves and adjust others’ perceptions about themselves.

Side effects resulting from prostate cancer treatment have received little attention regarding stigma, despite these potentially stigmatising side effects being well documented in quantitative and qualitative research (Broom, 2004, 2009; Chapple and Ziebland, 2002; Kelly, 2009; Korfage et al., 2006; Mols et al., 2009; Oliffe, 2009a). Side effects are common outcomes of all major primary treatments for prostate cancer (Chen et al., 2009). For UI, one of the most common treatment side effects, there has been little sociological attention as to how this condition may or may not be stigmatising. Fergus et al. (2002) has identified an ‘invisible stigma’ for men experiencing erectile dysfunction (ED) resulting from prostate cancer treatment. This refers to the shame men feel about their ED but also the fact that the condition cannot be seen by others and so is kept ‘invisible’. Importantly, the onset of ED is recognised as posing a threat to men’s masculine identities.

Much academic literature on UI comes from clinical, nursing, and psychological journals, with minimal sociological research exploring the condition, particularly as a treatment side effect for prostate cancer. Urinary incontinence (UI) can lead to a loss of the ‘physically bounded body’ (Lawton, 1998: 131) and consequently is symbolically loaded with meaning. Becoming incontinent as an adult can indicate frailty, loss of social capability (Isaksen, 2002; Mitteness and Barker, 1995) and cause feelings of embarrassment and shame to the sufferer (Eisenhandler, 1993). For men, UI poses challenges to historically rooted assumptions that men should have control over their bodies (Jervis, 2001) and it has been argued that men are more heavily reliant on being continent than women (Morgan, 1993).

UI has been identified as a stigmatising condition for men following treatment for prostate cancer (Paterson, 2000), yet this study was comprised of themes from just three interviews. Some different techniques to cover stigma and pass as normal have been identified for men with UI more broadly, such as wearing dark clothing that disguises incontinence (Elstad et al., 2010). However, a gap remains to explore how men experience and manage their UI as an iatrogenic side effect of prostate cancer treatment.

## Masculinities, embodiment and chronic illness

An embodied approach to masculinities can provide a fuller understanding of men’s lived experiences. Bodies have long been understood to be the central and dominant sources from which masculinities ‘proceed’ (Connell, 2005: 45).

Connell’s (2005: 61) notion of ‘body-reflexive practices’ conceptualises bodies as both objects *and* subjects of social practice. Social processes and historical forces in part produce bodies and yet bodies are still material and the way that bodies are used in practice ‘shape[s] the structures within which bodies are appropriated and defined’ (Connell, 2005: 61). Connell’s (2005) approach here offers a way of theorising masculinities as being *embodied*.

Crossley’s (2006, 2007) concept of ‘reflexive embodiment’ bears similarities to Connell’s (2005) ‘body-reflexive practices’. These are explicated by discussing two key aspects of both theories: *body techniques* and *reflexivity*. ‘Body techniques’ are bodily actions that are historically and culturally produced, where people’s perceptions and assessments of each social encounter contribute to an ordering of each encounter that makes sense of it and follows a pre-established pattern of behaviours that are specific to local social spaces and understood by each member of the social encounter as being appropriate to that encounter. Each time these behaviours are enacted they are reproduced and perpetuated, explaining how micro structures of social interaction play a significant role in shaping and reproducing historically and culturally situated body techniques, while recognising the possibility of change and embodied human agency as playing a part in shaping the process. This theorisation addresses the sociological dilemma of the relationship between agency and structure. Our understandings of established patterns of behaviour in different social contexts assists us as we creatively, and sometimes innovatively, negotiate and accommodate our way through physical spaces and social interactions in our everyday lives. Further to this understanding, Crossley (1995) draws on Goffman’s (1971) work *Relations in Public*. Within this work, Goffman observes that body techniques that are exercised in public are not just reproducing a practical order but ‘equally a moral order’ (Crossley, 1995: 140). It is important for people to demonstrate to others that they are of ‘sound character’ (Crossley, 1995: 140) and because of this there is a desire to maintain routinised patterns of behaviour within appropriate settings so that people can show their soundness of character to others by behaving normally. Consequently ‘body techniques, in this respect, are oriented towards a moral order which they both respect and reproduce’ (Crossley, 1995: 140).

The second similarity, *reflexivity*, is termed ‘body-*reflexive* practices’ by Connell (2005) and ‘*reflexive* embodiment’ by Crossley (2006). Connell’s concept has been described further above. Crossley’s (2006) is concerned with how we learn in childhood to perceive ourselves as objects, but only historically (as ‘me’), while the active part of who we are, the ‘I’, is forever in the present. The active ‘I’ and the passive ‘me’ are evident in the way that people talk about themselves and their bodies. People engage in work upon their bodies and such work is done to maintain or modify the body in some way, such as ‘I wash myself’, which Crossley uses to show how ‘body work is reflexive work, work on the body by the body’ (Crossley, 2006: 105) and such actions can be understood as *reflexive body techniques* (RBTs) (Crossley, 2006, 2007).

To form new reflexive body techniques (RBTs), close and constant monitoring of the body must first be undertaken (Schrock and Boyd, 2006). RBTs have been identified as being disseminated through interactions with others and therefore emerge and take hold throughout a society via social networks. In this way, some RBTs can be more widespread and common, while others can be rarer and more specialised. RBTs reflect the culture in which they are formed and applied. They can constitute such mundane behaviours that sociologists can fail to treat them as social objects worthy of sociological inquiry, yet the patterns and trends of such behaviours can provide rich insights into the values of a society. Crossley’s (2006, 2007) notion of reflexive body techniques (RBTs) provides a tool that, while similar to Connell’s (2005) notion of ‘body-reflexive practices’, is more conceptually developed and offers greater explanatory power.

**Figure 1.**
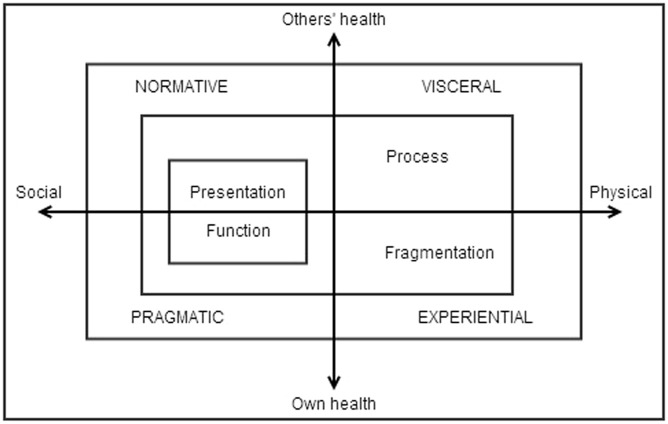
Watson’s model of ‘being in shape’: An embodied concept of masculinity. Source: Watson (2000: 116).

Watson’s (2000) empirically informed model of ‘being in shape’, predicated on Connell’s theory of masculinities, adds to the conceptual toolkit already outlined by conceptualising men’s embodied relationships with masculinity and health. Watson’s (2000) model of ‘being in shape’ is comprised of three components: ‘managing ambiguities’, a ‘male body schema’ and ‘evaluating social fitness’. ‘Managing ambiguities’ draws on the idea that masculinity is a dynamic ‘going concern’ that is a constant process of negotiation for men (115). Watson’s ‘male body schema’, shown in Figure 1, is a unifying theory to comprehend the different levels of embodiment that men occupy. Just as there are different masculinities (Connell, 2005), Watson asserts that masculinities are embodied in various ways between a person and their environment. Masculinities are embodied at the ‘normative’, ‘pragmatic’, ‘experiential’ and ‘visceral’ levels. Bodies are ‘presentational’, constituting symbolic modes for transmitting cultural and social values regarding masculinities and health (normative embodiment). Pragmatic embodiment is primarily functional, where men construct bodies in relation to having a ‘normal everyday body’ necessary for fulfilling specific gendered functions (e.g. ‘father’), and is the ‘primary site for interaction between social structure and practice’ (Watson, 2000: 119). Experiential embodiment is where the boundaries of the social and physical touch, in the experience of emotions and fleeting moments we are conscious of the predominantly hidden visceral processes (e.g. pain). Lastly, visceral embodiment is the indirect and non-consciously experienced grounding of the body in the world. These are the modes through which men’s bodies are experienced and discursively produced, useful for framing an understanding of how men engage with and experience the world.

‘Evaluating social fitness’ is the recognition of other healthy bodies through identifying how others present themselves, primarily concerning their ‘fitness’. For the men in Watson’s study, fitness was more important than health, and fitness constituted the capability to perform everyday gendered roles. Watson (2000: 122) summarises this as the ‘everyday function = masculine = fitness’ equation. To be masculine under Watson’s framework, men must be sufficiently fit to fulfil everyday tasks, illustrating that the pragmatic mode of embodiment is the most important for men. Men measure their masculinity by the tasks and functions that they perform and this in turn is a demonstration of their fitness to others.

Robertson et al. (2010) draw on theories described above to show how experiential embodiment is expressed through pragmatic embodiment during the process of ‘getting back to normal’ in a cardiac rehabilitation clinic, where men were encouraged to engage in a programme of exercise or yoga. Within men’s accounts, a ‘vibrant physicality’ (Monaghan, 2001) was expressed in relation to exercise and a ‘relaxed physicality’ in relation to yoga. Most men opted for exercise, following perceived gendered expectations of appropriate bodily activities for men, despite a recognition that ‘relaxed physicality’ was important to cardiac recovery. Notions of fitness could be emphasised by discussing the ‘vibrant physicality’ of exercising and by describing the effects of exercising on the physiological processes of the visceral body. Such talk was part of attempts to renegotiate embodiment through adopting a ‘new outlook’ on life, including a concern with ‘relaxed physicality’ to manage stress, which is associated with the risk of future cardiac events. Robertson et al. (2010) draw from these findings that recovery regimens need to contextually address not only the physical functional needs of individual men but also their emotional needs. Furthermore, they find that addressing men’s emotional needs requires a ‘pragmatically embodied ‘action’ component’ rather than just ‘talking therapies’ (Robertson et al., 2010: 701).

Charmaz’s (1994, 1995) work examines how the onset of chronic illness impacts on men’s masculine identities and completes the conceptual toolkit by providing a bridge between masculinities and moral order. The impact of trying to hide illness, particularly for men in seeking to preserve their public identities, can be damaging over long periods (Charmaz, 1994, 1995). Charmaz (1995: 268) has asserted that:

Illness can reduce a man’s status in masculine hierarchies, shift his power relations with women and raise his self-doubts about masculinities.

The onset of chronic illness can pose a range of ‘identity dilemmas’ for men: the dilemma of either ‘risking activity’ or being resigned to ‘forced passivity’, of ‘remaining independent’ or ‘becoming dependent’, of ‘maintaining dominance’ or ‘becoming subordinate’ and of ‘preserving public persona’ or ‘acknowledging private feelings’; whichever ways men choose to direct themselves in relation to these dilemmas there is always a cost to them (Charmaz, 1994).

In trying to live normal lives, men will devote considerable time and energy to strategies of ‘controlling time, pace, space, information and people’ to preserve their sense of self in the wake of chronic illness (Charmaz, 1994: 282). This paper will examine men’s UI experiences and management strategies in relation to the ‘identity dilemmas’ and strategies for control that Charmaz identifies.

Using the theoretical tools for understanding masculinities provided by Connell (2005), Watson (2000) and Robertson (2006), recognised as having compatible frameworks for unitary analysis (Robertson et al., 2010), offers a means of exploring how the experience and management of treatment side effects are related to men’s embodied masculinities. Additionally, Crossley’s (2006) notion of ‘reflexive embodiment’ provides an explanatory framework for understanding embodied practices while recognising the ‘moral order’, an area that requires attention due to the focus on examining stigma in relation to UI experiences and management strategies. Lastly, Charmaz’s (1994, 1995) work provides an intersectional framework for understanding how men respond to threats to their masculine identity and of breaching the moral order due to illness. This research asks *what are men’s experiences of UI and how do they manage these?* This informs a broader question, addressed from different perspectives in other works (Green 2021a, 2021b) of *how do men maintain their masculinity following treatment for prostate cancer?*

## Research design

A purposive sample (Bryman, 2016) of men who had received treatment for prostate cancer was selected. Voluntarily organised prostate cancer support groups (PCSGs) were selected as recruitment sites. Support groups are sites where information and experiences are shared and can sometimes be emotionally challenging (Ussher et al., 2006), which can facilitate the acquisition of richer data. Two prostate cancer support groups (PCSGs) in the Southeast of England were selected, following email and then telephone contact with the chairmen of each group. Both chairmen served as gatekeepers, supporting communication with their respective memberships by providing study information through email mailing lists and by introduction at an in-person steering group meeting for one of the groups. The ethical approach was developed in accordance with university ethical guidelines and received favourable ethical approval prior to commencing fieldwork from the author’s departmental ethics committee.

A qualitative, open interviewing approach was employed, using a topic guide to provide some commonality between interviews. The flexibility accommodated by open interviewing facilitated addressing sensitive topics, by being able to return to them over the course of the interview and approach them from different angles (Fielding and Thomas, 2008; Mason, 2002).

Interviews began with broad questions about employment, family life and general health, to ease participants into the interview and to encourage them to talk freely and without much prompting (Oliffe and Mroz, 2005). Masculinity can be a barrier to data collection within interviews (Schwalbe and Wolkomir, 2002) and young interviewers have been found to lack a more conversational style and be less willing to share their own experiences within interviews (Manderson et al., 2006), which can encourage men to be active in interviews and break away from a medical consultation model of health talk (Oliffe, 2009b). I adopted a respectful, deferential attitude by giving interviewees space at the beginning of interviews to speak without extensive probing or querying, with an open and conversational approach where I offered my own stories and reflections about my grandfather’s prostate cancer experiences, which inspired this research.

The age range for the men interviewed was between 53 and 83 years, with more than 60% of the sample being in their late sixties or early seventies. More than half the sample had a radical prostatectomy as their primary treatment and just under half had radiotherapy either as a primary or secondary treatment. All the men interviewed were White British or White European. The interviews generally lasted between 1 and 1.5 hours.

More than half the sample had received primary treatment within the last 5 years at the point of an interview, with the longest interval since primary treatment being fifteen years, although many interviewees received secondary prostate cancer treatments and other procedures for managing treatment side effects. Collected data are subjective reflections on past events, relying on recollections framed through interviewees’ current situations, a key limitation of the study. Participants frequently kept health records of their cancer experiences and these were directly referred to as reminders during interviews. Also, by virtue of regularly attending PCSGs, their accounts often appeared well-rehearsed through repeated telling in these spaces.

A thematic approach to coding was undertaken, where data collection and analysis led to the identification of common themes that guided later interviews and contributed to a data-driven analytical process. A qualified and constructivist grounded theory approach (Charmaz, 2014) was adopted to guide thematic analysis, whereby the construction of data is understood as a joint process of meaning making, and data are interpreted and re-interpreted over the course of the research. Interpretation and analysis of data is accordingly ongoing throughout data collection, as part of a reflexive analytical process. The ‘qualified’ component of the approach means that rather than a strictly inductive approach to coding, analysis was guided by an initial literature review and questions were generated to address topics of interest identified because of this literature review and from pilot interviewing. Initially, open coding (Corbin and Strauss, 2008) was undertaken to identify common themes based on the meanings that participants attached to topics they spoke about. Themes were coded and tested as to whether they were common across all interviews and whether emerging patterns were consistent throughout the sample, using the constant comparative method (Charmaz, 2014). In the process of identifying and testing emerging themes, analytical notes were taken as markers to return to, to remind me of my thinking at different stages during the analysis, and to suggest possible links in the data between themes that might contribute to the development of theory. Over the course of the analysis, codes were subdivided into more specific themes and were sometimes merged when themes overlapped, and the software Nvivo 10 facilitated this process. Analysis was undertaken by the author with review and discussion with my PhD supervisors. Participants’ accounts are presented below with pseudonyms, providing their age at the point of interview and abbreviations for their treatment(s) (see Supplemental Appendix 1).

## Experiences of urinary incontinence

UI may never occur for men treated for prostate cancer or may occur but then cease entirely shortly following treatment, yet for more than half of the men interviewed urinary problems continued to varying degrees to be a concern for extended periods of time following treatment. Most of the men interviewed described being aware that UI could be a treatment side effect, although this was being reported with hindsight. For a broader exploration of men’s decision-making for treatment with consideration to the wider context concerning prostate cancer and other symptoms please see Green (2021a). UI could pose significant challenges to how men went about their everyday lives.

The men who experienced UI were very concerned with the shame and embarrassment that passing urine in public would cause them. Nigel recalled an episode when the bag for his urinary sheath catheter (a device for collecting leaking urine) broke in a supermarket.

***Nigel***

*I used to like wearing shorts and then, can’t wear shorts (because of urinary sheath catheter), so used to wear the cut-off jeans, so you’ve got enough fabric there to cover your bag, and all that sort of thing, but then you’re out, I always remember I was dressed up like that and we went shopping in Sainsbury’s (supermarket) and the bloody bag split . . . my wife just, she was picking stuff off the shelves one minute, and then I was gone, I just ran . . . I phoned her on her phone, I said “I’m back in the car”, she said “what are you doing”, I said “the bloody bags split”, “oh Christ”, but that’s sort of, that’s embarrassing level, because nobody knew quite . . . what’s happened, ‘he’s wet himself’, you know, ‘what’s up with him, is he some sort of drunkard or something?’ (laughs)*

(67, RARP)

Nigel changed his usual style of dress to hide his urinary sheath catheter from others in public. His account shows a concern that others would see him as disreputable, as ‘some sort of drunkard’. Geoff was also very concerned about others noticing his incontinence in public.

***Geoff***

*You become hyperaware of the fact that you are leaking, and it’s probably brought about by the sort of the public perception of elderly smelly men and women who urinate a bit, and you’ve seen these people in the street, you know, people who are elderly and have an incontinence problem usually smell, it’s just how it is . . . and unless you’ve got a good regime of pads, it does- and a good regime of showers and what have you, that smell stays with you*

(63, RP, RT, HT)

Geoff pays particular attention to the smell of urine and his concern with masking the smell ‘that stays with you’. He also draws on a broader social stereotype, which other men drew on as well, of ‘elderly smelly men and women’ with who they associated public episodes of incontinence. Nigel and Geoff’s accounts demonstrate experiences of ‘felt stigma’ (Scambler, 2009) for their UI, feeling shame or fear of potentially experiencing enacted stigma, where discrimination occurs against the social unacceptability of a person’s stigmatising condition (Scambler, 2009). Neither of these men makes any reference to being discriminated against by others, yet both of their accounts show considerable efforts to avoid incontinent episodes being witnessed in public, either by running to the car or by a routine of frequent washing. Experiences of felt stigma for UI pose challenges for men in how they go about their everyday activities in public and still maintain their masculinity in doing so.

## Management strategies for urinary incontinence

### Monitoring the body

Two of the most common methods of managing UI were to wear incontinence pads or to wear a urinary sheath catheter over the penis to collect leaking urine into a bag, usually tied around the leg or ankle. Monitoring the body is important with either method in going about day-to-day living for these management strategies, whether that is in public or private. Clive used incontinence pads and describes the difficulties he encountered when going about domestic activities in and around his house, while Nigel describes the difficulties of using an external sheath catheter at his workplace.

***Clive***

*The end of the male urethra is not fixed like it is with a woman, it moves around, and it’s alright when I’m sat down, sedentary . . . the bladder fills up, and then you can go to the toilet, but if you’re outside, like occasionally I am, if- or on the computer and moving around and I’m in the garden and mowing or cutting or doing, once you start to concentrate on living, you forget, and occasionally, you know if you’re under a car or you’re moving around, then your body moves and your clothes move with you, and occasionally, you can find yourself leaking, just outside the area, which, believe it or not, it can happen, and so, um, occasionally I get caught out, um, I would be ill advised I think to go more than four hours without checking my pad*

(76, RP, RT)

***Nigel***

*if you’re doing any stretching about, that sort of thing, that’s where I’d be careful, climbing up ladders and squeezing through gaps, if you stretch your leg and then all of a sudden something goes pop (referring to urinary sheath catheter and bag), and you go “oh no!”*

(67, RARP)

The necessity for Clive to monitor himself is clear from the ‘ill advised(ness)’ of going longer than four hours without checking his pad. Monitoring the body is shown to be a conscious, regulatory act, for when he starts ‘to concentrate on living’ he is liable to ‘forget’ about his pad and consequently finds himself ‘leaking’. Nigel, too, must be ‘careful’ in undertaking the once taken-for-granted physical activities required for his paid employment. The bodily practices that Clive and Nigel had previously taken for granted in going about daily mundane tasks become problematised with UI and now require constant attention by monitoring the body to try and avoid leaking that can occur when attention slips. Schrock and Boyd (2006) have identified monitoring as a precursor before adopting new ‘reflexive body techniques’ (RBTs; Crossley, 2006) in response to a desire to either maintain or modify the body.

Clive and Nigel’s accounts also illustrate how managing UI with incontinence pads or urinary sheath catheters relies to a large extent on men limiting their movements and strenuous exertions. ‘Pragmatic embodiment’ is the primary mode for demonstrating masculinity, where it is important for men to be physically fit enough to fulfil and perform gendered functions (Watson, 2000). For chronically ill men, one of the core dilemmas that they face in their masculine identity is ‘risking activity’ or instead being forced into passivity (Charmaz, 1994). For Clive and Nigel, engaging in everyday activities had become problematised and to risk engaging in activity required paying close attention by monitoring their leaking bodies.

Geoff and Dan’s accounts further above and below describe their reliance on daily incontinence pads to manage their UI. They describe monitoring for the feeling of dampness on their skin, checking by touch whether pads were damp and smelling for the scent of urine. Failure to identify bodily signs would pose the threat of experiencing enacted stigma. Covering incontinence from others in public by engaging in constant monitoring put a strain on men’s daily lives, evident in Dan’s description of how his routine was drastically adapted by the need to check and change his incontinence pads ‘six or seven times a day’.

***Dan***

*I used women’s incontinence pads, they just happened to fit in my brand of underwear, that I wanted still to wear, umm, relatively unobtrusively. Meant that I had to change six or seven times a day*

***Interviewer***

That’s quite an undertaking

***Dan***

*Oh it is, but, I never went anywhere without a man-bag with three sets of pants and Christ knows how many pads, like with your bag, mine would have been filled with underwear and pants.*

(66, RP)

Dan’s account illustrates the extent of effort exerted to be able to ‘pass’ as normal in daily public life. To manage this strain, Dan made some concessional changes to how he went about managing his incontinence. Here Dan describes how he changed his regular exercise routine from working out at the gym to swimming.

***Dan***

*I can say that swimming was great, the best exercise to do in front of people, because nobody knows you’re incontinent . . . when you’re swimming you’re flat in the water you’ll never leak, once you stand upright you can feel yourself possibly leaking, but seriously though you’re just peeing down into the water. But for anyone who wants to do exercise, it gets- the whole of you is wet, so if you did dribble a bit, nobody’s going to know, but it’s those silly practicalities of life that make things bearable*

(66, RP)

Swimming was an ideal activity for Dan, in that it enabled him to preserve important aspects of his masculine identity through doing it. He was able to remain active, be independent and preserve his public persona, all being important ‘identity dilemmas’ for men facing chronic illness (Charmaz, 1994). Furthermore, Dan is still able to show his continuing physical fitness and functioning, demonstrating his pragmatic embodiment (Watson, 2000) and continuing masculinity despite the difficulties of his UI. By changing his regular exercise method and wearing women’s incontinence pads, Dan made concessions that threatened his masculine identity but were done to maintain his capability of being active, independent, dominant, and to preserve his public persona (Charmaz, 1994), as well as to continue fulfilling gendered functions (Watson, 2000). This was true of other men managing UI with incontinence pads or urinary sheath catheters, where concessions in their activities were made to preserve masculine identity more broadly. Managing identity dilemmas in the wake of UI puts considerable strain on men who try to continue exhibiting the masculine values Charmaz (1994) describes. The extent of the strain is likely to vary by stage in the life course, where requirements for public activity will differ for those in paid employment compared to those who have retired.

Paying close and constant conscious attention through *monitoring* the leaking body is the primary means by which these men managed identity dilemmas and preserved their masculine identity. However, when the strain of constant monitoring was too exhausting, men sought to make concessions to their condition in their conduct of daily activities.

### Planning for public encounters

Travelling in public was a particularly important concern for men and managing incontinence while travelling required preparing in advance, as Algernon describes.

***Algernon***

*We had a family gathering at the end of November . . . so I had one of these milk churns in the car, in case of, which I had to use, not to drink milk, but- (both laugh)*

(73, RT)

The benefit of having a car allowed Algernon to travel and manage his continence with less chance of experiencing enacted stigma because of the relatively private space that a car affords when travelling in public. This is further demonstrated in Clive’s account below.

***Clive***

*I’m in John Lewis’ restaurant, and their toilet was in the adjacent side of the floor, and I said “I need to go”, and I got up to walk about, and I had completely voided (emptied his bladder) by the time I got there . . . we went out and we got a pair of trousers and underpants, as a back-up, in the boot of the car, so . . . I changed in the car park, in the back of the car, we’ve got frosted windows, which I hadn’t particularly wanted, but became a great benefit*

(76, RP, RT)

Prior to treatment most of the men in this research had no impediments to their mobility, were physically active, and engaged in public activities how and when they pleased without concern. Moderate or severe UI changed this, displacing them from public spaces they had once felt comfortable in due to the perceived risk of disrupting the ‘moral order’ of interactions in these spaces (Goffman, 1971). As certain public spaces became associated with the feelings of fear and shame of felt stigma, with a perceived increased likelihood of enacted stigma occurring, men sought to avoid such spaces or find ways of traversing them with relative security.

Beyond travelling by car, further planning and preparation were required to engage in activities in public. Dan described how going shopping in his local town centre had become difficult because of the frequency and urgency with which he had to urinate. To help manage this, Dan had come to know every toilet in the town so that he would not be caught out by his incontinence. Andy, too, had to be prepared when going to the cinema, as he describes:

***Andy***

*I go (to the toilet) before I go in, and this is it you see, I go to the loo here, and when I get to the cinema I nip in and I don’t really (need to use the toilet), but I nip in just to be safe, then before I come home, it depends, if I think oh I can make it home alright its fine, then I don’t, but, so that’s the only downside, you’re always thinking ahead*

(68, RTwHT, RP)

The ‘downside’ of Andy’s strategy was his having to pay considerable conscious attention to his body by ‘always thinking ahead’. Persistent experiences of UI constitute an ongoing concern for managing the possibility of incontinence occurring whenever going out in public, requiring not just *monitoring* but also *planning*.

### Disciplining the body

Following the onset of UI, men commonly sought to undertake strategies that would reduce and completely stop their incontinence. One of these strategies was to undertake pelvic floor exercises to strengthen the muscles that control urination, as Arnold describes.

***Arnold***

*I persisted with the pelvic floor exercises and it gradually got better, and now it’s, probably 95% ok . . . I persisted with the pelvic floor exercises for months and months and months, until I was almost normal*

(83, RP)

Arnold’s dedication in persisting ‘for months and months’ with pelvic floor exercises illustrates how some men would be willing to submit themselves to new disciplined physical routines to improve their continence.

***Chris***

*I told myself that the sooner that I could stop using pads the greater that the, not desire, the greater the possibility of me being able to sort out incontinence problems, because I said to myself that, I would be forced- with pads there’s always the reassurance that it’s there, and therefore if you leak it doesn’t matter too much, you’ve just got to change the pad when you get home, if you’re out, but if you don’t have the pad then there’s a greater incentive to try to control things*

(73, RP)

For Chris, the use of incontinence pads partly constituted a concession to his body being limited by UI. By removing the safety barrier of incontinence pads, Chris ‘forced’ himself into improving his continence with the ‘incentive’ of negative consequences of the enacted stigma he would face if he was incontinent in public to motivate him.

Both Arnold and Chris’ accounts suggest that it was their desire to return to normal and their force of will that was key to their success and tell how they have reclaimed control over their bodies, thereby maintaining masculinity. A benefit to undertaking pelvic floor exercises is evident from Robertson et al.’s (2010) cardiac rehabilitation study, where they conclude that a ‘pragmatically embodied ‘action’ component’ is important to men’s recovery regimens. While pelvic floor exercises may not have masculine signifiers, indeed may have more feminine connotations, they have a ‘vibrant physicality’ (Monaghan, 2001; Robertson, et al., 2010) that involves *doing* something as an active approach to recovery by disciplining the body. In contrast to *monitoring* and *planning*, which are reactive approaches to managing unruly bodies, which as described above can involve minimal movement to avoid spillage and therefore a ‘relaxed physicality’ (Robertson et al., 2010). Unfortunately, men who attempted pelvic floor exercises would often give them up in the course of time with limited or no improvement, resigning themselves to having to wear incontinence pads or urinary sheath catheters, to *manage* rather than *recover* from their UI.

## Discussion

Men were found to experience felt stigma for their UI but rarely enacted stigma (Scambler, 2009). Feelings of fear and shame associated with felt stigma shaped how they approached managing their UI and how they maintained their masculinity in doing so. This is evident across the three strategies of *monitoring, planning*, and *disciplining* identified in these findings. An understanding that there is an inherent interdependence between managing felt stigma for UI and maintaining masculinity emerges, each is shaped and responds to the other.

*Monitoring* the body was necessary when managing UI with incontinence pads and urinary sheath catheters. These methods relied upon minimising bodily movement and strenuous effort, running counter to the physically active primary ‘pragmatic’ mode of male embodiment (Watson, 2000). This posed the ‘identity dilemma’ of either ‘risking activity’ or accepting ‘forced passivity’ (Charmaz, 1994). To ‘risk activity’, close and constant monitoring of leaking bodies was required, putting a strain on men’s lives. To manage this strain some men curtailed or changed activities, accepting some loss of masculine identity to preserve masculinity more broadly.

*Planning* for public encounters was another important strategy. Engaging in public with UI could contribute to feelings of felt stigma and posed a perceived threat to the ‘moral order’ (Goffman, 1971) of public relations. Maintaining masculinity necessitates the ongoing performance of gendered functions (Watson, 2000), which frequently requires men to go into the public sphere for everyday living. Engaging in public space is particularly important for maintaining masculine identity following onset of UI, as it is key to all four of Charmaz’s (1994) masculine ‘identity dilemmas’. Being able to ‘preserve public persona’ requires a demonstration of normalcy in public and engaging in regular activities in public is a way of demonstrating ‘activity’, ‘independence’, and ‘dominance’ to others and to oneself.

*Disciplining* the body was a strategy that differs from *monitoring* and *planning* as it was oriented towards active *recovery* rather than reactive *management* of a persistently unruly body. The importance of having an ‘action’ component to men’s recovery regimens (Robertson et al., 2010) is a consideration that offers opportunities across different recovery contexts for men’s illness experiences. Including an ‘action’ component in recovery support interventions could enhance men’s recovery experiences and improve recovery outcomes and this warrants further research attention.

Examining the strategies that men employed to manage their UI has provided a set of possible factors that may influence the adoption of new reflexive body techniques. The first factor, informed by Schrock and Boyd’s (2006) assertion of the necessity for close and constant monitoring of the body as a prerequisite for RBT formation, is the degree of routineness that a bodily concern must be attended to. Urination occurs so frequently that it is easier to form new management strategies for UI and sustain them over time, as the regularity of urination lends itself to the formation of new habitual behaviours. The second factor is the desire or motivation to address a bodily concern. There is a strong desire to not experience felt or enacted stigma, so men adopt management strategies to reduce feelings of the former by reducing the likelihood of the latter. The third factor is the degree of unruliness of the body, the extent to which the body will comply with management or recovery strategies. This paper finds the experience of UI being a position well-disposed towards adopting new reflexive body techniques in relation to the first two of these three points, in terms of desire for maintaining masculinity and not breaching the ‘moral order’ and in terms of routine by how frequently urination occurs. With a greater understanding of the mechanisms that are facilitators and barriers to adopting techniques that support active recovery strategies, this paper offers guidance for how recovery interventions can be designed to better support men to engage with them in ways that align with their masculine identities, and therefore which they are more likely to positively engage with. Further, this paper illustrates how failure to design recovery interventions with masculinity in mind can impact on men’s post-treatment experiences, particularly for stigmatising conditions like UI, where men who cannot manage their UI reduce their engagement with public life.

This research study has limitations in several aspects of its design. Men were recruited through two PCSGs in the South East of England. Men’s post-treatment UI experiences who do not attend PCSGs are not explored here and may differ for those with different demographic characteristics. Recruiting from support groups can lead to a sample skewed towards certain demographic characteristics, such as men who are white, well-educated, and nearing retirement (Breau and Norman, 2003; Broom, 2009), which mirror the sample for this paper. The role of the support group is discussed in other works (Green, 2021a, 2021b). Motivation to attend a PCSG may suggest unmet emotional or social needs, illustrating how a prostate cancer diagnosis presents multidimensional unmet needs, in a context where many men will not receive any support beyond their clinical care. Further attention is needed to explore how embodied emotionality can be recognised and addressed in recovery interventions, following prostate cancer treatment and for other forms of recovery.

Recruiting from support groups also had strengths. Having attended support group meetings before, men were open to talking about their experiences, likely more so than they might have been had they not attended a support group before. Further, exploring men’s experiences at varying intervals following treatment offered insights into men’s changing experiences and evolving management strategies over time, in seeking to understand the chronic dimensions of post-treatment experiences. Lastly, the homogeneity of the sample provides a stronger basis for making claims about the specific group of men that was recruited for this research.

## Conclusion

This paper contributes to a growing understanding of the relationship between morality and older men’s masculinities, where interdependence has been described between managing felt stigma for UI and maintaining masculinity. Three strategies of *monitoring, planning* and *disciplining* were identified and described. *Monitoring* and *planning* being are associated with *reactive management* of UI, particularly important for engaging in public space, and demonstrating activeness in public life, an important aspect of maintaining masculinity. *Disciplining* is associated with *active recovery*, acting on the body to change it, rather than manage its unruliness. Three factors are posited as components for adopting new reflexive body techniques: (1) routine (how frequently the technique is performed, (2) desire (the will for change), and (3) unruliness (the body’s non-compliance with the technique, or otherwise). This paper finds the experience of UI being a position well-disposed towards adopting new body techniques in relation to the first two of these three points, in terms of desire for maintaining masculinity and not breaching the ‘moral order’ and for routine by how frequently urination occurs. The third factor varied considerably among the men experiencing UI, where for some men their situation improved whereas for others their actions had limited to no effect on the state of their UI.

## Supplemental Material

sj-docx-1-hea-10.1177_13634593231185266 – Supplemental material for Experiences and management of urinary incontinence following treatment for prostate cancer: Disrupted embodied practices and adapting to maintain masculinity Supplemental material, sj-docx-1-hea-10.1177_13634593231185266 for Experiences and management of urinary incontinence following treatment for prostate cancer: Disrupted embodied practices and adapting to maintain masculinity by Richard Green in Health
