# Supplementary material for: Experiences and management of urinary incontinence following treatment for prostate cancer: Disrupted embodied practices and adapting to maintain masculinity
Source: Health (London). 2023 Jun 30;28(4):489–506. doi: 10.1177/13634593231185266 (PMC11151700; doi:10.1177/13634593231185266)
Supplement: sj-docx-1-hea-10.1177_13634593231185266 – Supplemental material for Experiences and management of urinary incontinence following treatment for prostate cancer: Disrupted embodied practices and adapting to maintain masculinity [file sj-docx-1-hea-10.1177_13634593231185266.docx]

**Appendix 1: List of Abbreviations**

ED Erectile Dysfunction

HT Hormone Therapy

PCSG Prostate Cancer Support Group

RARP Robot-Assisted Radical Prostatectomy

RBT Reflexive Body Technique

RP Radical Prostatectomy

RT Radiotherapy

RTwHT Radiotherapy with Hormone Therapy

UI Urinary Incontinence
